# Analysis of Residual DSBs in Ataxia-Telangiectasia Lymphoblast Cells Initiating Apoptosis

**DOI:** 10.1155/2016/8279560

**Published:** 2016-01-06

**Authors:** Teresa Anglada, Mariona Terradas, Laia Hernández, Anna Genescà, Marta Martín

**Affiliations:** Departament de Biologia Cel·lular, Fisiologia i Immunologia, Universitat Autònoma de Barcelona, Edifici C, Bellaterra, 08193 Cerdanyola del Vallès, Spain

## Abstract

In order to examine the relationship between accumulation of residual DNA double-strand breaks (DSBs) and cell death, we have used a control and an ATM (Ataxia-Telangiectasia Mutated) defective cell line, as Ataxia-Telangiectasia (AT) cells tend to accumulate residual DSBs at long times after damage infliction. After irradiation, AT cells showed checkpoint impairment and a fraction of cells displayed an abnormal centrosome number and tetraploid DNA content, and this fraction increased along with apoptosis rates. At all times analyzed, AT cells displayed a significantly higher rate of radiation-induced apoptosis than normal cells. Besides apoptosis, 70–85% of the AT viable cells (TUNEL-negative) carried ≥10 *γ*H2AX foci/cell, while only 12–27% of normal cells did. The fraction of AT and normal cells undergoing early and late apoptosis were isolated by flow cytometry and residual DSBs were concretely scored in these populations. Half of the *γ*H2AX-positive AT cells undergoing early apoptosis carried ≥10 *γ*H2AX foci/cell and this fraction increased to 75% in late apoptosis. The results suggest that retention of DNA damage-induced *γ*H2AX foci is an indicative of lethal DNA damage, as cells undergoing apoptosis are those accumulating more DSBs. Scoring of residual *γ*H2AX foci might function as a predictive tool to assess radiation-induced apoptosis.

## 1. Introduction

Following DNA double-strand breaks (DSBs) generation by ionizing radiation (IR), the cell undergoes an Ataxia-Telangiectasia Mutated (ATM) dependent p53 activation of the DNA damage response (DDR) cascade to activate the cell checkpoints in order to gain time for DNA repair [1–3]. If the DNA damage cannot be repaired during checkpoint arrest, cells are driven to undergo an irreversible fate by apoptosis or senescence [4, 5]. Sensing of the DNA damage involves the extensive phosphorylation of histone H2AX molecules at both sides of the DNA break [6]. Phosphorylated H2AX forms foci immediately after DNA damage induction by IR. These *γ*H2AX IRIF (Ionizing Radiation-Induced Foci) are detectable with immunostaining or cytometry techniques as soon as 3 minutes afterwards, and the maximum number of foci is detected 30–60 minutes after irradiation [7]. The number of *γ*H2AX foci has been found to closely correlate with the number of radiation-induced DSBs [8, 9]. Very soon after irradiation, *γ*H2AX foci are numerous and small and they disappear along with resolution of DNA damage [8]. Nonetheless, several results have shown that radiosensitive cell lines retain *γ*H2AX foci longer than radioresistant cell lines after exposure to radiation [10–12]. The fraction of tumor cells that retain *γ*H2AX foci 24 hours after irradiation has been correlated with the fraction of cells that fail to divide and form colonies [13, 14], suggesting that the H2AX assay can be used as an indicator of cell death. However, there are also negative studies that found no correlation between *γ*H2AX and clonogenic cell survival [15, 16], demonstrating that it is yet unclear whether residual DSBs are ultimately related with apoptosis triggering.

Recently, apoptosis and mitotic catastrophe (MC) have been functionally linked [17, 18]. MC has been defined as an oncosuppressive mechanism resulting from a combination of deficient cell cycle checkpoints, persistence of DNA damage and mitotic failure, which can ultimately lead to cell death during mitosis or alternatively by apoptosis or senescence. This mechanism mainly operates in a cell-cycle-specific kinases-dependent and p53-dependent way to avoid accumulation of genomic instability and is prevalent in cancer cells that exhibit genomic instability and are devoid of proper checkpoint control [17, 19, 20].

The goal of this study was to examine the relationship between DNA damage accumulation and apoptosis. In radiosensitive ATM deficient cells, the defects in triggering the whole DDR following IR lead to checkpoint failure and to the accumulation of unresolved DSBs [1, 3], thus being an ideal model to study this relationship. Our results show that AT and normal lymphoblastoid cell lines undergoing apoptosis accumulate a higher number of *γ*H2AX foci than those belonging to the viable fraction. Specifically, AT lymphoblasts accumulate a higher fraction of residual DSBs and undergo significantly higher levels of IR-induced apoptosis at all postirradiation (pIR) times analyzed. Also AT lymphoblasts display a strong G2/M arrest and tetraploidization, suggesting that MC contributes to apoptosis in ATM deficient cells.

## 2. Results and Discussion

### 2.1. AT Lymphoblasts Efficiently Trigger a p53-Dependent Apoptotic Response and Undergo High Levels of Radiation-Induced Apoptosis

In order to determine a relationship between persistent radiation-induced DSBs and apoptosis, we had to first determine the ability to undergo apoptosis in AT and normal lymphoblastoid cell lines (LCLs). To this end, Annexin-V (An) and propidium iodide (PI) incorporation in cells was measured by flow cytometry, and cells were analyzed at 0, 24, 48, and 72 hours after 5 Gy irradiation. Loss of plasma membrane asymmetry by exposing phosphatidylserine in the outer leaflet is an early event in the apoptotic process, previous to loss of membrane integrity and to DNA fragmentation. Thereby, cells positive for Annexin-V and negative for PI staining (An+/PI−) are considered to be undergoing early stages of apoptosis (EA) [21–23]. Later in this process, cells lose membrane integrity, allowing PI staining. Therefore, cells that are An+/IP+ are considered to be in late apoptosis (LA), although necrotic cells can also be found in this fraction [21, 24].

**Figure 1:**
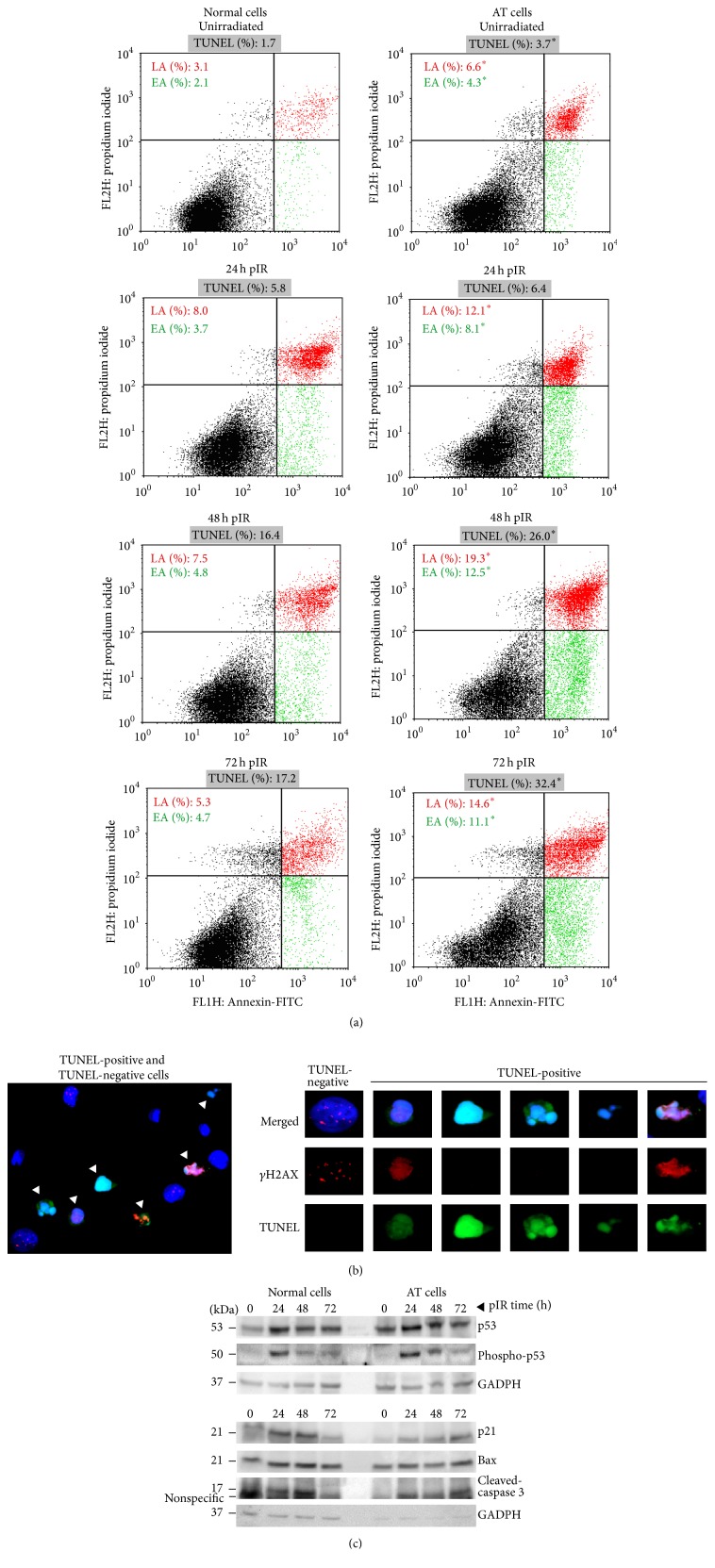
(a) Radiation-induced apoptosis measured by means of Annexin-V/PI and TUNEL methodologies. Cytometry plots were used for gating cells stained using Annexin-V (An) and propidium iodide (PI) before and after irradiation. In all plots, the lower left quadrant corresponds to the viable, nonapoptotic cell population (An−/PI−). The lower right quadrant corresponds to the cell population An+PI–, which is undergoing early apoptosis (EA) and is shown in green. The upper right quadrant corresponds to the cell population An+PI+, which is undergoing late apoptosis (LA) and is shown in red. Frequencies of EA and LA are shown in each graph at 0, 24, 48, and 72 hours after irradiation in normal and AT cells and they correspond to the mean of 3 different experiments with two replicas each. A minimal number of 10000 cells were analyzed in each experiment. The asterisks indicate statistical differences in the apoptotic levels between AT and normal cells when comparing the EA fraction, the LA fraction, and the sum of Annexin-V-positive cells (EA + LA). In all cases, *χ* ^2^ test was applied and the *p* values were <0.005. Frequencies of TUNEL-positive cells for each cell type at 0, 24, 48, and 72 hours pIR are shown over each cytometry plot. The asterisks indicate statistical differences between AT and normal cells (*χ* ^2^ test; *p* values < 0.007). The values for TUNEL were obtained after scoring 1000 cells for each time point and each cell line using an epifluorescence microscope. (b) Scoring of TUNEL-positive cells. On the left, a general view under the microscope (40x) showing irradiated cells in which a combination of TUNEL staining (green) and *γ*H2AX immunofluorescence (red) has been applied. DNA is stained with DAPI (blue). TUNEL-positive cells (white arrowheads) depict intense TUNEL staining and they show the morphological features of apoptotic cells (right panel): smaller nuclei with highly condensed chromatin—intensely stained with blue—undergoing variable levels of nuclear fragmentation. Also, TUNEL-positive cells could depict a pan-nuclear *γ*H2AX staining but never had *γ*H2AX foci. (c) Western blot detection of apoptotic markers. Normal and AT cells were irradiated with 5 Gy of *γ*-rays and expression of p53, its activated form phospho-p53 (Ser15), and other p53 targets such as p21, Bax, and the cleaved fraction of caspase 3 were analyzed at 0, 24, 48, and 72 hours after irradiation. Proteins were detected in two different experiments and GADPH was used as the housekeeping gene.

As shown in Figure 1(a), the fraction of An+/PI− cells is higher in AT LCL at all times analyzed, even before irradiation. AT cells undergoing early apoptosis reach 8.0% and 12.5% at 24 hours and 48 hours pIR, respectively, while the fraction of normal cells An+/PI− during these time points is always lower than 5%. The overall fraction of Annexin-positive cells (sum of An+/PI− and An+/PI+ cells) reaches its maximum at 48 hours after irradiation, being 12.3% in normal LCL and 31.8% in AT LCL. At later time points, An+/PI− and An+/PI+ cells start to slowly decline, although they are still significantly higher in AT cells and far from the basal levels in both cell lines. These results demonstrate that this AT LCL not only efficiently triggers apoptosis, but also undergoes higher rates of radiation-induced apoptosis than its normal counterpart.

Because several works have reported contradictory results regarding apoptosis induction in AT cells [25–31], we aimed to confirm the previous results obtained with Annexin-V/PI by analyzing radiation-induced apoptosis using TUNEL methodology. One characteristic feature of the later stages of apoptosis is the internucleosomal fragmentation of DNA into ~180 bp repeats often referred to as DNA laddering [32]. TUNEL allows the detection of these DNA fragments by labeling their 3′-OH end with a fluorescent molecule. AT and normal lymphoblasts were irradiated at the same dose of *γ*-rays (5 Gy), and apoptosis levels were analyzed at the same time points previously described. TUNEL was performed on slides and quantitation of TUNEL-positive cells was performed with an epifluorescence microscope. The majority of TUNEL-positive cells also displayed characteristic morphological features of apoptosis, such as nuclei shrinkage, DNA compaction, and nuclear fragmentation. All these features combined with TUNEL staining allowed certain detection of apoptotic cells (Figure 1(b)).

As shown in Figure 1(a), the levels of spontaneous apoptosis measured with TUNEL were higher in AT than in normal cells. Higher rates of spontaneous apoptosis in AT lymphoblasts have been described before [33] and are confirmed by the results obtained in the present study with both apoptotic cell detection assays (total Annexin-V-positive cells before irradiation: 5.2% in normal cells versus 10.9% in AT cells; *χ* ^2^ test, *p* < 0.0001; TUNEL: 3.7% in AT cells versus 1.7% in normal cells; *χ* ^2^ test, *p* < 0.0072). At early postirradiation times, the fraction of TUNEL-positive cells remains low in both cell lines, but they increase at 48 hours pIR and reach maximum levels at 72 hours pIR, being of 17.2% in normal and 32.4% in AT cells (*χ* ^2^ test, *p* < 0.0001). Although both Annexin-V/PI and TUNEL methodologies measure apoptosis, they seem to detect correlative stages of this process. At twenty-four hours after irradiation, there has been an increase of cells undergoing EA and evolving to a LA stage compared to unirradiated cells, while yet very few cells are positive for TUNEL staining. EA and LA fractions reach a* plateau* level at 48 hours pIR, while at this time there is an increasing frequency of TUNEL-positive events. Because TUNEL methodology detects extensive DNA fragmentation, TUNEL-positive cells might undergo a later apoptotic stage than those signaled with Annexin. In this way, the combination of the results obtained with the Annexin-V/PI and the TUNEL procedures renders a dynamic picture of the apoptotic process in the lymphoblast cells analyzed.

Lymphocytes are removed, both physiologically and after irradiation, by a p53- and caspase-dependent apoptotic pathway that leads to DNA cleavage [19, 34, 35]. The role of the ATM protein in triggering this IR-induced apoptotic response has been examined using different experimental systems in AT lymphoblasts, AT lymphoblastoid cell lines (LCLs), and Atm^−/−^ mouse thymocytes with conflicting results. Lymphocytes from AT patients were found to have an increased spontaneous apoptotic level [33]. Also, a normal apoptotic response after IR was demonstrated in Atm^−/−^ mouse cells [26] and in lymphocytes from AT patients [27]. Variable results have been described in AT LCLs, although most of them displayed a normal apoptotic response to IR [28, 36]. To ultimately determine p53 status, we analyzed p53 presence and its activation after IR. Levels of p21, a p53 effector involved in cell cycle arrest at G1 and S phases after DNA damage induction [37], have also been analyzed. As shown in Figure 1(c), despite ATM absence, p53 was effectively induced in normal and AT cells at 24 hours pIR, when the fraction of apoptotic cells starts to increase. Consistent with greater apoptotic induction, levels of activated p53 are still high in AT cells at 48 hours pIR. Induction of p21 is observed in both cell lines although higher expression is observed in normal than in AT cells. In this regard, it has been suggested that ATM regulates distinct p53-dependent pathways that selectively trigger checkpoint arrest or apoptosis. For example, effective p53 induction coupled with checkpoint failure and a normal apoptotic response after IR has been described in ATM deficient cells [26, 28, 38, 39]. In agreement with these works, normal cells efficiently arrest at G1 after irradiation while the AT lymphoblastoid cell line tested in this study undergoes high apoptosis rates along with G1 checkpoint failure (see Section 2). Bax, another p53 target involved in activation of caspases, shows a similar expression in both LCLs. The cleaved fragment of caspase 3 is detected only after irradiation in both cell lines but in AT cells its expression is still visible at 72 hours, consistent with higher frequency of apoptotic AT cells at this time point. Altogether, our results are in agreement with a role for ATM selectively activating p53 to regulate cell-cycle checkpoint but not apoptosis. In this regard, ATM- and Rad3-related (ATR), Chk2 and DNA-PKcs have been proposed as candidates to regulate IR-induced apoptosis in AT cells [38–40].

### 2.2. Radiation-Induced Mitotic Catastrophe Is a More Relevant Cell Death Process in AT Lymphoblasts Than in Its Normal Counterparts

**Figure 2:**
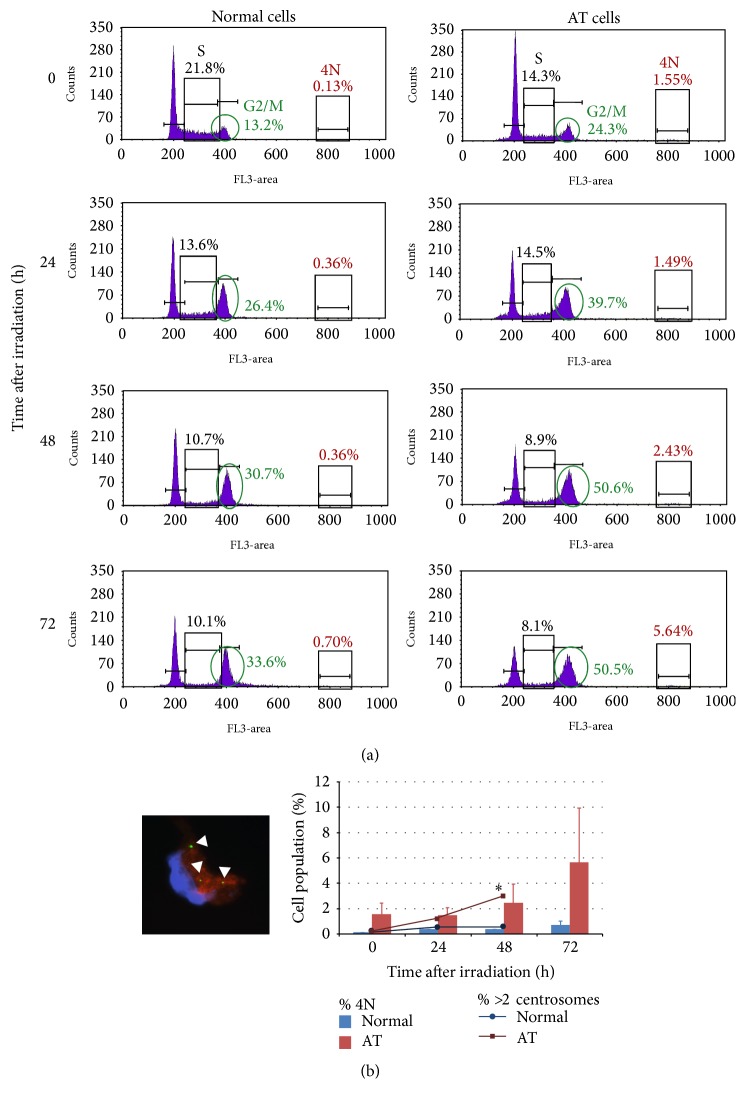
(a) Cell cycle analysis. The histograms show the cell cycle distribution of normal and AT cells before irradiation and at 24, 48, and 72 hours after irradiation. Cell cycle distribution was obtained by means of PI staining, which measures DNA content. The frequency of cells entering in S-phase for each cell type and each time point is shown, evidencing lack of IR-induced G1 checkpoint arrest in AT cells. The fraction of cells arrested in G2/M after irradiation and the tetraploid population (4N) arising after irradiation have also been highlighted. The frequencies displayed are the mean of two independent experiments in which a minimum number of 10000 cells were analyzed. (b) Tetraploidization and centrosome number. The image shows an AT lymphoblast (probably a metaphase) with 3 pericentrin signals (green; white arrowheads). The DNA is stained with DAPI and the red staining corresponds to *α*-tubulin. The bars in the graph show the fraction of tetraploid cells scored in AT and normal lymphoblasts before and after irradiation. The values are the mean of two experiments, and the error bars show the standard deviation. The lines in the graph depict the fraction of cells with an abnormal centrosome number (>2) within the same time points. The values for centrosome number were obtained after analyzing a minimal number of 400 cells for each cell type and each time point. The asterisk indicates statistical differences between normal and AT lymphoblasts in the frequency of cells with more than 2 centrosomes (*χ* ^2^ test; *p* values < 0.002).

We proceeded by analyzing cell cycle progression after irradiation. As shown in Figure 2(a), normal lymphoblasts are efficiently arrested at G1, as demonstrated by a diminution of the S fraction at 24 h pIR that prevails up to 72 h. As expected, impairment of proper G1 arrest in the AT LCL was demonstrated by no noticeable decrease in the S fraction at 24 hours pIR, and later decreases were low when compared to normal cells. These results are consistent with the Western blot results showing a weak induction of p21 in AT cells after irradiation (Figure 1(c)) and are in agreement with the ATM deficiency cell phenotype, which is characterized by impairment of G1 and intra-S checkpoint activation upon DNA damage infliction. This leads to cell cycle progression of cells bearing unresolved DNA damage [3, 41]. These cells are efficiently arrested in G2 [42] unless the DNA damage has been inflicted during G2 phase, in which case ATM deficient cells proceed into mitosis [43]. In any case, damaged cells that surpass G1 and/or G2 checkpoints become later arrested in mitosis (M) because of spindle anaphase checkpoint (SAC). In this work, cells were irradiated during their exponential growth, implying that many AT cells will surpass G1 and intra-S checkpoints and will be arrested at G2, while those AT cells irradiated during G2 phase will surpass the G2 checkpoint and arrest in M by SAC. Remarkably, our results show that AT lymphoblasts significantly accumulate at G2/M after irradiation at all times analyzed (Figure 2(a)), suggesting that, besides cells arrested at G2 checkpoint, some of them might remain in M phase.

Persistent arrest at G2/M boundaries after DNA damage infliction is a first indicator of mitotic catastrophe (MC), so we aimed to analyze the possibility that MC contributes to cell death in AT cells. MC has been described as an oncosuppressive mechanism that, in order to avoid accumulation of genomic instability, senses this mitotic failure and responds to it by driving the cell to death during mitosis [20]. Sometimes, some of these cells do not die during mitosis and eventually overcome SAC signaling and “slip” into the next interphase without dividing. These cells may reenter the cell cycle and reduplicate its DNA content, turning into the accumulation of tetraploid cells within the population. Indeed, AT lymphoblasts showed an increasing frequency of cells with a 4N DNA content that reached 5.6% at 72 hours after irradiation (Figures 2(a) and 2(b)). Although small, this fraction was higher than in normal lymphoblasts, whose tetraploid population was lower than 0.7% at all times analyzed. To further confirm these results, we quantified the centrosome number, as cells that skip mitosis and reenter the cell cycle will reduplicate their centrosomes along with DNA. Centrosomes were scored by means of immunofluorescent pericentrin detection and cells were classified into those having a normal number of centrosomes (1 centrosome in interphase and 2 centrosomes in S, G2, and M phases) or an aberrant number of centrosomes (more than 2). As shown in Figure 2(b), the basal frequency of AT cells with >2 centrosomes was very low, but it strikingly increased at 48 hours after irradiation, when it reached almost 3% of the cell population and correlated with the appearance of a 4N cell population (Figure 2(b)). In contrast, the fraction of normal lymphoblasts with an abnormal number of centrosomes did not reach 1% of the population during the 48 h analyzed (*χ* ^2^ test, *p* < 0.002). Thus, some AT cells are able to reach mitosis despite defective repair. Eventually, some of them skip M phase, giving raise to the appearance of a tetraploid population together with an increasing population of cells with an abnormal centrosome number. Polyploid cells with extra centrosomes are prone to form transient multipolar mitotic spindles, which can either directly trigger mitotic death or result in the generation of aneuploid daughter cells. A small fraction of these cells might survive and enter a mitotic round that is also likely to be catastrophic [20]. Because the MC mechanism can ultimately culminate in apoptotic cell death [20], we propose that this mechanism contributes to the radiation-induced apoptotic levels detected in AT lymphoblasts.

### 2.3. Viable AT Lymphoblasts Display Higher Levels of Radiation-Induced DNA Damage and Delayed DSB Repair at Long Times after Irradiation

**Figure 3:**
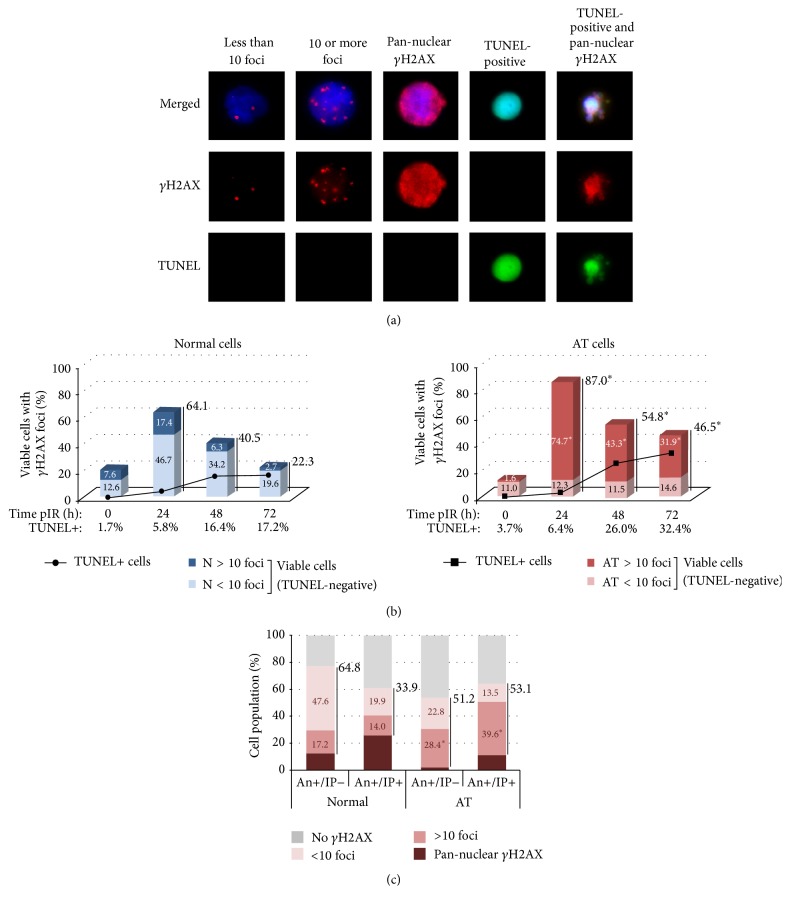
(a) Immunodetection of *γ*H2AX in lymphoblasts. DSBs were scored by *γ*H2AX foci detection in TUNEL-negative, An+/PI−, and An+/PI+ cells. Pan-nuclear *γ*H2AX staining was scored in TUNEL-negative, An+/PI−, and An+/PI+ and in TUNEL-positive cells. (b) *γ*H2AX-labeling in viable (TUNEL-negative) cells. The number and frequency of viable cells with *γ*H2AX foci are reflected in the bars. Within this fraction, the frequency of cells with <10 foci or with ≥10 foci is shown inside the bars. The asterisks indicate statistical differences between normal and AT lymphoblasts in the fraction of cells with *γ*H2AX foci or in the fraction of cells with ≥10 *γ*H2AX foci (*χ* ^2^ test; *p* values from *p* = 0.0270 to *p* < 0.0001). The frequencies for each category are calculated over the total number of TUNEL-negative scored cells. A minimal number of 350 TUNEL-negative cells were analyzed for each cell type and each time point. The apoptotic rate measured with TUNEL is depicted in the graph as a continuous line. Values for TUNEL-positive cells are given under the *x*-axis and are those corresponding to Figure 1(a). (c) *γ*H2AX-labeling in Annexin-positive cells. AT and normal cells were irradiated and fractions corresponding to EA and LA were isolated by cell sorting. An+/PI− and An+/PI+ cells were classified into those with or without *γ*H2AX foci and those with pan-nuclear *γ*H2AX staining. The frequency of cells with *γ*H2AX foci is depicted next to the bar. Within this fraction, the frequency of cells with less than 10 foci (light pink) or with 10 or more foci (pink) is shown inside the bars. The asterisks indicate statistical differences between normal and AT lymphoblasts in the frequency of cells with ≥10 *γ*H2AX foci (*χ* ^2^ test; *p* values from *p* = 0.0020 to *p* < 0.0001). The frequencies are calculated over the total number of An+/PI− and An+/PI+ sorted cells. A minimal number of 400 cells were analyzed for each cell type and each time point.

We next aimed to examine the levels of radiation-induced DNA damage in both lymphoblast cell lines. To this end, we analyzed *γ*H2AX foci corresponding to radiation-induced DSBs in viable cells—those negative for TUNEL (Figure 3(a)).  Figure 3(b) shows that, 24 h after irradiation, 64.1% of the normal lymphoblasts have *γ*H2AX foci, a 3.2-fold increase compared to unirradiated cells. Within the same time interval, viable AT lymphoblasts displayed a 7-fold increase, as 87% of them showed *γ*H2AX foci (*χ* ^2^ test, *p* = 0.0135). At 72 h pIR normal cells have repaired most of their DSBs and only 22.2% of them have *γ*H2AX foci, while this fraction is still around 50% in AT lymphoblasts (*χ* ^2^ test, *p* < 0.0001). All together, these results reflect the DNA repair impairment of AT cells, which repair most of the DSBs in a fast and efficient way, while a subset of breaks remains unrepaired for long times, even days, after DNA damage infliction [3, 10–12, 44]. In agreement with that, our results show that AT cells, despite showing an initial decline in the fraction of cells displaying *γ*H2AX foci, are unable to efficiently proceed to further diminish this population and accumulate high numbers of residual DSBs, even at very long times after DNA damage has been induced.

After that, we scored the number of *γ*H2AX foci in the TUNEL-negative cells and classify them into two groups: cells with less than 10 *γ*H2AX foci and cells with 10 or more *γ*H2AX foci (Figure 3(b)). Only a small fraction of the normal cells accumulated 10 or more *γ*H2AX foci, reaching a peak of ~18% at 24 hours pIR and declining thereafter. On the contrary, most of the irradiated AT cells accumulated 10 or more *γ*H2AX foci, reaching a maximum of around 75% at 24 hours pIR (*χ* ^2^ test, *p* < 0.0001). From 24 to 72 hours after irradiation, 85 to 70% of the *γ*H2AX-positive AT cells carry 10 or more DSBs while this frequency is much lower in normal cells (27 to 12%). Thus, after irradiation, AT cells accumulate more cells with DSBs and more DSBs/cell than their normal counterparts.

The results presented here suggest that DSB repair might be inversely correlated with apoptosis induction. Indeed, at 48 hours pIR, the percentage of normal cells with ≥10 *γ*H2AX foci is low (6.3%) and it coincides with the stabilization of the TUNEL-positive rate (around 17%). On the other hand, at 48 hours pIR, still most of AT cells have ≥10 *γ*H2AX foci (43.3%) and TUNEL rates continue to increase at 72 hours pIR (from 26 to 32%; Figure 3(b)). In agreement with that, recent studies have revealed that some residual 53BP1, Rad51, and *γ*H2AX foci remain in cells for a relatively long time after irradiation and have indicated an inverse correlation between the number of residual foci and the surviving fraction of cells [45–49]. Similarly, a correlation between a higher rate of foci loss and a higher clonogenic surviving fraction in ten different cancer cell lines has been described [50]. Finally, it is worth noting that the fraction of AT cells with less than 10 *γ*H2AX foci remains stable before and after irradiation and is hardly changed along with the apoptotic rate (Figure 3(b)), thus discarding this subpopulation of cells as that with more probabilities of undergoing IR-induced apoptosis. This result is in agreement with other studies describing that low background levels of foci (<3 foci per cell) scored at 24 hours after irradiation were correlated with cell survival [14, 51].

### 2.4. Apoptotic AT Cells Accumulate More Residual DSBs Than Normal Lymphoblasts

To further analyze this possibility, we proceeded to analyze radiation-induced DSBs in normal and AT lymphoblasts undergoing apoptosis. The characteristic DSB-signaling processes of the DDR, such as phosphorylation of histone H2AX forming visible foci, are eventually abolished in cells undergoing last stages of apoptosis, probably due to DNA condensation [52]. Consequently, no *γ*H2AX foci were scored in TUNEL-positive cells (Figure 3(a)). We reasoned that earlier apoptotic stages, such as those detected with Annexin-V/PI methodology, would better allow the detection of radiation-induced DSBs. To this end, AT and normal lymphoblasts were irradiated and enriched populations of early apoptotic (An+/PI−) and late apoptotic/necrotic (An+/PI+) cells were obtained by flow sorting at 48 hours after irradiation. Immediately after sorting, cells were fixed on slides and *γ*H2AX immunofluorescence was performed. This procedure resulted in the loss of Annexin-V and PI staining, allowing for reliable identification of *γ*H2AX signaling. In all the populations analyzed we found a fraction of cells displaying a pan-nuclear *γ*H2AX staining (Figure 3(a)). This kind of staining has been related to apoptosis induced by several DNA damaging agents and is concurrent with the initiation of DNA fragmentation resulting from the apoptotic process [53, 54]. This fraction of cells was taken into account when calculating the percentages shown in the figures. At the time point selected after irradiation (48 hours), the fraction of cells undergoing early and late apoptosis was at its maximum in both cell lines. Cells undergoing early apoptosis (An+/PI−) were 4.8% in normal and 12.5% in AT cells, while those undergoing late apoptosis (An+/PI+) were 7.5% and 19.3% in normal and AT LCL, respectively (Figure 1(a)).

Within the above mentioned fraction of cells undergoing early apoptosis (An+/PI−), most of them had *γ*H2AX foci—64.8% of the normal lymphoblasts and 51.2% of the AT cells (Figure 3(c)). Nonetheless, most of the cells undergoing early apoptosis had less than 10 *γ*H2AX foci/cell in normal cells (47.6%) but ≥10 *γ*H2AX foci/cell in AT cells (28.4%), demonstrating that also the AT cells that initiate apoptosis (An+/PI−) accumulate a significantly higher number of DSBs than normal cells (*χ* ^2^ test; *p* < 0.0001). As normal lymphoblasts enter in later apoptotic/necrotic stages (An+/PI+), the frequency of cells with less than 10 *γ*H2AX foci is sharply reduced (from 47.6% to 19.9%; 2.4-fold reduction), while the fraction of cells with ≥10 *γ*H2AX foci is more or less maintained (14%). Similarly, the frequency of AT lymphoblasts undergoing later apoptotic stages (An+/PI+) that present less than 10 *γ*H2AX foci is reduced, while the population of cells with ≥10 *γ*H2AX foci is increased with respect to early apoptosis and to normal cells (75% of the *γ*H2AX-positive AT lymphoblasts). Thus, AT lymphoblasts accumulate more DSBs/cell than their normal counterparts, also while undergoing apoptosis (*χ* ^2^ test; *p* = 0.0020).

It is important to note that An+/PI− cells might be considered viable, as early apoptosis is believed to be reversible if the conditions inducing apoptosis are removed [55–57]. Moreover, it has been suggested that DNA repair is involved in this reversibility [58]. Thus, while undergoing early apoptosis, cells might be able to perform some degree of DSB repair that leads to reduction of *γ*H2AX foci-positive cells in later apoptotic stages. Most probably, cells that carry a larger amount of DSBs have a lower probability of eventually performing successful repair; thus cells with ≥10 *γ*H2AX foci accumulate at later stages of apoptosis. In this work, cells undergoing late apoptosis are those accumulating more DSBs, and the cell line with impaired repair activity is the one carrying more residual DSBs and displaying higher rates of cell death. Similar results have been very recently described in which cells carrying Rad51 foci at 24 hours pIR are the ones more likely to die [44]. It cannot be concluded that accumulation of ≥10 *γ*H2AX foci signals cells to undergo apoptosis, as *γ*H2AX foci dose-response may depend on cell type, time allowed after irradiation, and the cell cycle phase in the moment of irradiation among other factors. Nonetheless, these results support the notion that persistence of residual DSBs signals those cells that are more likely to undergo cell death.

## 3. Conclusions

Annexin-V/PI and TUNEL methodologies have been used to analyze IR-induced apoptosis. These methodologies seem to detect subtle differences in apoptotic frequencies that might correspond to progressive apoptotic stages, as maximum levels of Annexin-V-positive cells are reached earlier than maximum levels of TUNEL-positive cells. Annexin-V/PI allows discrimination between cells undergoing early and late apoptosis as well as fast scoring of more cells. TUNEL detection on slides and subsequent microscope analysis allows the combination of TUNEL and protein immunodetection—like *γ*H2AX—and the analysis of these events in the apoptotic and the healthy fraction at the same time.

The results in this work show that AT LCL efficiently undergoes IR-induced apoptosis to a higher level than its normal counterpart at all times analyzed. Along with accumulation of residual DSBs, indicators of mitotic catastrophe such as prolonged G2/M arrest and DNA and centrosomes reduplication are found in irradiated AT cells, which also contribute to the apoptotic levels scored. In these cells, apoptosis is accompanied with p53 induction and cleavage of caspase 3, while they show low levels of p21 induction that correlate with impairment of G1 and intra-S checkpoint activation after irradiation. These results agree with a role for ATM selectively activating p53 to regulate apoptosis and not cell cycle checkpoint. Accumulation of radiation-induced unrepaired DSBs contributes to cell death. For this reason, viable cells that are undergoing apoptosis (Annexin-positive) have been isolated in both cell lines and *γ*H2AX foci have been scored. The results show that these are the cells accumulating more DSBs per cell. As cells progress from EA to LA, the fraction of cells with fewer *γ*H2AX foci decreases in both AT and normal cells, while cells with more than 10 *γ*H2AX foci accumulate in LA, especially in AT cells—consistent with their DNA repair defect. These results support the notion that persistence of residual DSBs signals those cells that are more likely to undergo cell death and that scoring of *γ*H2AX foci might function as a predictive tool to assess radiation-induced apoptosis.

## 4. Material and Methods

### 4.1. Cell Culture and *γ*-Irradiation

EBV-transformed human lymphoblastoid cell lines (LCLs) GM08436A (derived from a child suffering from Ataxia-Telangiectasia) and GM09622 (derived from a sex and age matched control) were obtained from the Coriell Cell Culture Repositories. Cells were grown in suspension in RPMI 1640 medium with GlutaMAX-I (Life Technologies, CA, USA) supplemented with 15% fetal bovine serum and kept in the incubator at 37°C and 5% CO_2_ atmosphere. When indicated, cells were irradiated with 5 Gy *γ*-rays using an IBL-437C R-137 Cs irradiator, with a dose rate of 5.10 Gy/min.

### 4.2. Apoptosis Detection

#### 4.2.1. Annexin-V-Fluorescein Assay

The Annexin-V/propidium iodide (PI) assay was performed following the manufacturer's instructions (Annexin-V-FLUOS Staining Kit, Roche, Switzerland). Briefly, lymphoblast cells were collected, centrifuged, and washed in 1xPBS. The cell pellet was resuspended in freshly prepared Annexin-V-FLUOS labeling solution with PI and incubated for 15 minutes (min) at room temperature, in the dark. Cells were analyzed on a FACSCalibur flow cytometer (Becton-Dickinson, CA, USA) using 488 nm excitation and 530/30 nm band pass filter for fluorescein detection and a FL2 photomultiplier and band pass filter 585/42 nm for PI detection after electronic compensation. Flow cytometry analysis was done with the CellQuest software (Becton-Dickinson, CA, USA). Cells were classified into the following fractions: (a) viable cells (An−/PI−) were impermeable for PI and also did not bind Annexin-V (An); (b) early apoptotic cells (An+/PI−) bound An and were PI impermeable; (c) late apoptotic or, also called, secondary necrotic cells (An+/PI+) bound An and were PI permeable; (d) primary necrotic cells (An−/PI+) only displayed PI staining. When indicated, cell sorting of enriched populations of An+/PI− and An+/PI+ cells was performed with a FACS Aria SORP sorting cytometer (Becton-Dickinson Biosciences, CA, USA) using a 488 nm excitation laser and 525 nm band pass filter for fluorescein detection and a 350 nm excitation UV laser and 660/400 nm band pass filter for PI detection. Cells obtained after sorting were dropped on poly-L-lysine coated slides and allowed to attach for 3 min before proceeding with immunofluorescence.

#### 4.2.2. TUNEL Assay

The TUNEL assay was performed following the manufacturer's instructions (In Situ Cell Death Detection Kit, Fluorescein, Roche, Switzerland). Briefly, lymphoblasts were centrifuged, washed with 1xPBS, and dropped on poly-L-lysine coated slides. Cells were then fixed with 2% paraformaldehyde for 20 min at room temperature and permeabilized with 0.1% Triton-X100 and 0.1% sodium citrate in 1xPBS for 5 min in ice. The TUNEL mix was applied to the cells following the manufacturer's instructions and allowed to be incubated at 37°C for 40 minutes. Before analysis, Vectashield Mounting Medium for fluorescence (Vector Laboratories Inc., CA, USA) supplemented with 4′,6-diamino-2-phenylindole (DAPI) was applied. TUNEL analysis was performed with an Olympus BX41TF epifluorescence microscope equipped with an Olympus U-TVIX digital camera using the Isis v5.4.9 software (MetaSystems, Germany).

### 4.3. Cell Cycle Analysis

Lymphoblasts were washed in 1xPBS, centrifuged, and kept in 70% ethanol at −20°C until analysis. Cells were then centrifuged, washed with 1xPBS, and resuspended in 1 mL of freshly prepared PI/Triton/RNAsa solution: 0.1%Triton-X100, 2 mg RNAsa A (DNAsa free), and 200 *μ*L of 1 mg/mL PI. After 15 min incubation at 37°C, DNA content was measured on a FACSCalibur flow cytometer (Becton-Dickinson, CA, USA). Red fluorescence of PI-stained nuclei was excited at 488 nm with an argon laser and was collected through a 670 nm long pass filter detection into the FL3 photomultiplier tube on a linear scale, at a flow rate of 12 mL/min (low). Cell cycle analysis of the DNA histograms of integrated red fluorescence was performed with CellQuest software (Becton-Dickinson, CA, USA).

### 4.4. Immunofluorescence

Immunodetection of *γ*H2AX was performed on the same samples previously analyzed for TUNEL or sorted after Annexin-V/PI staining. Pericentrin detection was performed on newly obtained samples of irradiated lymphoblasts allowed to attach onto poly-L-lysine slides. Cells were fixed for 15 min in 4% paraformaldehyde and permeabilized in 1xPBS-0.5% Triton-X100 solution for 15 min. After 30 minutes of blocking with 0.1% Tween20 and 5% FBS, mouse anti-*γ*H2AX (Ser139) (Upstate/Millipore, MA, USA) or rabbit anti-pericentrin (Abcam, UK) was applied at a 1 : 1000 concentration and allowed to incubate for 1 hour at room temperature. Anti-mouse Cy3 (Amersham Biosciences/GE Healthcare, NJ, USA) and anti-rabbit A488 (Invitrogen/Molecular Probes, OR, USA) secondary antibodies were applied at 1 : 1000 final concentration for 45 minutes at room temperature, followed by extensive washing. Before analysis, Vectashield Mounting Medium for fluorescence (Vector Laboratories Inc., CA, USA) supplemented with DAPI was applied. Slides were analyzed using an Olympus BX41TF epifluorescence microscope equipped with an Olympus U-TVIX digital camera using the Isis v5.4.9 software (MetaSystems, Germany).

### 4.5. Western Blotting

Cells were collected by centrifugation, washed with PBS, treated with RIPA lysis buffer, and sonicated. Whole-cell extracts were loaded onto a 10% SDS-polyacrylamide gel that was run at 150 V for 50 minutes in a Bio-Rad mini-gel system. Proteins were transferred to a nitrocellulose membrane (30 V, 90 minutes) and blocked for 1 h at room temperature in 5% BSA brought to 37°C or with nonfat milk at 4°C. Primary antibodies used were mouse monoclonal anti-p53 (Santa Cruz Biotechnology, Inc., TX, USA), rabbit monoclonal anti-phospho-p53 (Ser15) (Thermo Fisher Scientific, MA, USA), rabbit monoclonal anti-p21 (Abcam, UK), rabbit monoclonal anti-Bax (Abcam, UK), rabbit polyclonal anti-active caspase 3 (Abcam, UK), and mouse anti-GAPDH (Abcam, UK). Membranes were washed with TTBS (Tris 10 mM, NaCl 150 mM, and 0.005% Tween20) and incubated for 1 hour at room temperature with secondary antibody, goat anti-rabbit or goat anti-mouse, conjugated to horseradish peroxidase (Upstate/Millipore, MA, USA). Proteins were visualized using Immobilon Western kit (Upstate/Millipore, MA, USA) and the signal was captured with ChemiDoc XRS (Bio-Rad, CA, USA).

### 4.6. Statistical Analysis

The statistical analysis was performed using GraphPad InStat version 3.05 (GraphPad Software Inc., CA, USA).
